# An Institutional Mechanism for Assortment in an Ecology of Games

**DOI:** 10.1371/journal.pone.0023019

**Published:** 2011-08-05

**Authors:** Paul E. Smaldino, Mark Lubell

**Affiliations:** 1 Department of Psychology, University of California Davis, Davis, California, United States of America; 2 Department of Environmental Science and Policy, University of California Davis, Davis, California, United States of America; Hungarian Academy of Sciences, Hungary

## Abstract

Recent research has revived Long's “ecology of games” model to analyze how social actors cooperate in the context of multiple political and social games. However, there is still a paucity of theoretical work that considers the mechanisms by which large-scale cooperation can be promoted in a dynamic institutional landscape, in which actors can join new games and leave old ones. This paper develops an agent-based model of an ecology of games where agents participate in multiple public goods games. In addition to contribution decisions, the agents can leave and join different games, and these processes are de-coupled. We show that the payoff for cooperation is greater than for defection when limits to the number of actors per game (“capacity constraints”) structure the population in ways that allow cooperators to cluster, independent of any complex individual-level mechanisms such as reputation or punishment. Our model suggests that capacity constraints are one effective mechanism for producing positive assortment and increasing cooperation in an ecology of games. The results suggest an important trade-off between the inclusiveness of policy processes and cooperation: Fully inclusive policy processes reduce the chances of cooperation.

## Introduction

This paper develops an agent-based model of cooperation in multiple public goods games (PGGs). The model operationalizes Long's [1] notion of an “ecology of games,” which recognizes that humans are social animals that interact in many different social settings. For example, an individual in a community may be simultaneously involved in school, work, church, social organizations, and political groups. Membership in each institution is not mutually exclusive, and some of the same constellations of individuals may be in a number of institutions. The ecology of games is relevant for collective and political decision making as well, because political actors must divide their time among a wide range of decision processes for any particular issue area [2]. Developing models that account for the ecology of games facing individuals is thus an important goal for understanding complex social phenomena.

Our model includes social processes and institutional arrangements that endogenously structure [3] how individuals participate in multiple games, and can cluster actors in ways that favor cooperative strategies. Our approach builds on two families of models that have been developed in the literature on multiple games and cooperation: 1) models that rely on fixed spatial or network connections (or fixed degree distributions) to structure interactions [4]–[13]; and 2) models where game strategies and network structures co-evolve on unipartite networks without a fixed number of links [14]–[17].

We consider a third type of model, which features a bipartite network of actors and games in which the actions of leaving current games and joining new games are de-coupled. The bipartite network is not equivalent to the unimodal networks commonly used to study multiple game settings. The unimodal projection of a bipartite network has edges that capture the number of shared games, but does not account for heterogeneity among games. For example, actors A and B might share two games, one game with actor C and the other with actor D. A unipartite project would treat the two interactions between A and B as equivalent and ignore the potentially important consequences of a different third player in each game. Our model also differs from several mentioned above [5], [6], [9], [12], [14]–[16] in the use of multiplayer (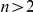) games.

We make an additional contribution by considering minimally simple institutional mechanisms for assortment. Specifically, we show that capacity constraints on the number of players allowed in a single game makes it more likely that cooperative strategies will interact together. Such simple institutional mechanisms may be easier to implement than more complex institutional and individual mechanisms for producing cooperation [18], including punishment [19], [20] and reputation [4], [21]. Complex institutions may incur a higher level of transaction costs that must be subtracted from the overall social gains from cooperation. Hence, from a comparative institutional analysis perspective, it is generally preferable to choose simpler institutions that provide greater net benefits.

The capacity constraint institution is not equivalent to Olson's [22] famous hypothesis about group size, which focuses on the decreasing marginal returns of individual contributions to a public good. In contrast, the capacity constraint mechanism limits group size in a way that promotes cooperative assortment–the tendency for cooperative individuals to associate at a rate higher than chance [23], [24]. The result thus generalizes to the multiple game setting the finding from single game studies that positive assortment and clustering of cooperative strategies is a necessary condition for the evolution of cooperation [25], [26]. We argue that any institutional mechanism that promotes positive assortment will have a similar result, and suggest comparative institutional analysis should the balance the speed at which assortment occurs with the costs of institutional design.

It is important to note that our model differs from dynamic evolutionary models in which strategies compete for reproductive success. Here, strategies are fixed, and we focus on institutional mechanisms by which extant cooperators may assort. Two points should be made about this. First, in classic evolutionary game theory [26], [27], a mean advantage for cooperators is a necessary condition for cooperation to evolve. Second, an underlying assumption of our model is the capacity for a large amount of social reorganization to occur between generations of reproduction (or imitation). This has previously been shown to benefit cooperation in evolutionary models [6], [9]. It is therefore useful to study the relative payoffs and social reorganization in a population of fixed-strategy agents. Future versions of this model will more explicitly explore the feedbacks between the time scale of social reorganization and evolutionary strategy selection.

## Methods

### A Basic Model for an Ecology of Public Goods Games

The PGG is a common model for representing interactions in which the group benefits from individual contributions, but individuals do better by free-riding. The rational solution to a single-round game is therefore to contribute nothing, though positive contributions can be maintained by mechanisms of reputation [4] or punishment [19], [20]. In the absence of secondary mechanisms, spatial clustering can promote cooperation in the PGG with evolutionary replicator dynamics [4], [7].

We consider a population of individuals each playing one or more PGGs, such that individuals may have overlapping co-players in different games. The games structure the interactions between individuals, and that structure can change as individuals leave and join games.

Individuals have fixed resources 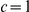 to contribute every time step, and each agent either defects by failing to contribute to any games in which the individual is participating, or cooperates by contributing all of its resources, divided evenly among all the games in which the individual is participating. In real-world interactions, not every individual has the same resources, nor would they necessarily contribute equally to all games. Our main motivation for using fixed resources was to develop our initial model using simple assumptions. The decision was also motivated theoretically by the finding that fixed resources promote the evolution of cooperation in agent-centered public goods games on a heterogeneous network [7], and is also a common investment heuristic [28].

After each round of game play, some individuals have the opportunity to join new games or leave current games. When joining games, they have information about how much a given game paid out to its participants, but not what each player contributed. When leaving games, individuals only know what they personally received. Joining and leaving are independent, and some games may become “dead” if all players leave.

**Figure 1:**
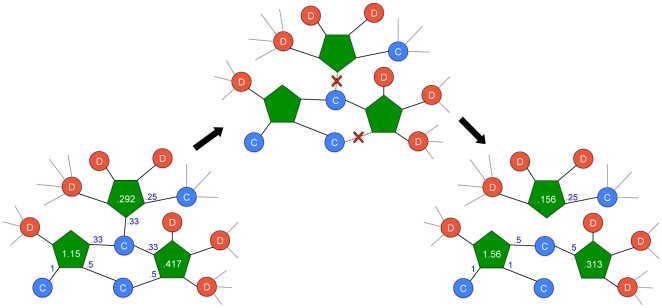
Example model dynamics. Player contributions (cooperators only) to each game are indicated in blue at the end of the links connecting players to games, with 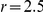. Each public goods game (green pentagons) pays out 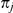 to each of its players, as indicated in white. Players may then attempt to join or leave games. Cooperator-heavy games are much more stable. For illustrative convenience, in this example none of the agents shown join a new game, and all agents are allowed to leave the games which are shown. For the individuals playing games not shown in the figure (grey links), it is assumed that either those agents were not chosen to attempt to join or leave this turn, or that they were unable to join any new games and that their offscreen games all yielded payouts greater than their contributions.

The agent considers all games with positive payouts and attempts to join one with probability proportional to its relative payout. Agents may then attempt to leave a game. An example of this aspect of the model dynamics is shown in Figure 1. The agent considers all its current games, excluding any just joined, and leaves the one with the lowest payout if and only if that payout is less than or equal to the individual's contribution to that game. In other words, individuals only leave games that don't yield positive returns. The process is continued until an equilibrium is reached in which all movement is stops, usually within a few hundred time steps (see Figure S3).

### Institutional Constraints

In addition to the basic model, we examine two institutional constraints that may structure the ecology of games, which we call *budget constraints* and *capacity constraints*. Budget constraints refer to limitations on the number of games in which an individual can participate, and may be conceived of as the limitations on a person's available time, capital, or cognitive resources.

Capacity constraints refer to limitations on the number of individuals that can participate in a single game. Institutions may have formal restrictions on membership numbers, such as a limited number of seats on a board, or restrictions may be informal, such as a maximum number that can effectively communicate in a group.

### Model Specifics

A population of 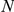 agents play pure strategies of cooperate or defect, where 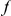 is the frequency of cooperators, and are initially distributed among 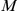 public goods games. Budget constraints may enforce a maximum number of games per player, 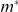. Similarly, capacity constraints may impose a maximum number of players per game, 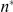. Agents are initially placed in 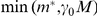 games, and placement ends if all games reach 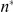 players.

Each time step, all active public goods games are played. The per-agent payout for each game 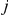 is
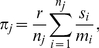
(1)where 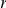 is the game production and 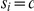 if 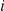 is a cooperator and zero if 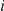 is a defector. Each agent then has the opportunity to try and join a new game, with probability 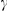, , where 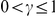 is a constant that determines the rate of agent “social mobility.” Individuals are restricted to games being played by a current co-player, which represents the fact that information about the existence of social opportunities spreads through networks [29]. The agent considers all games with positive payouts and attempts to join one with probability proportional to its relative payout. Agents then attempt to leave a game, again with probability 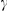 (Figure 1). The agent considers all its current games, excluding any just joined, and leaves the one with the lowest payout if and only if that payout is less than or equal to the individual's contribution to that game. In other words, individuals only leave games that don't yield positive returns. The process is continued until an equilibrium is reached. Our results are averaged from 100 runs of each condition, and unless otherwise stated, we used values of 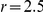, 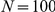, 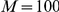, 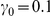, and 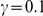.

## Results

**Figure 2:**
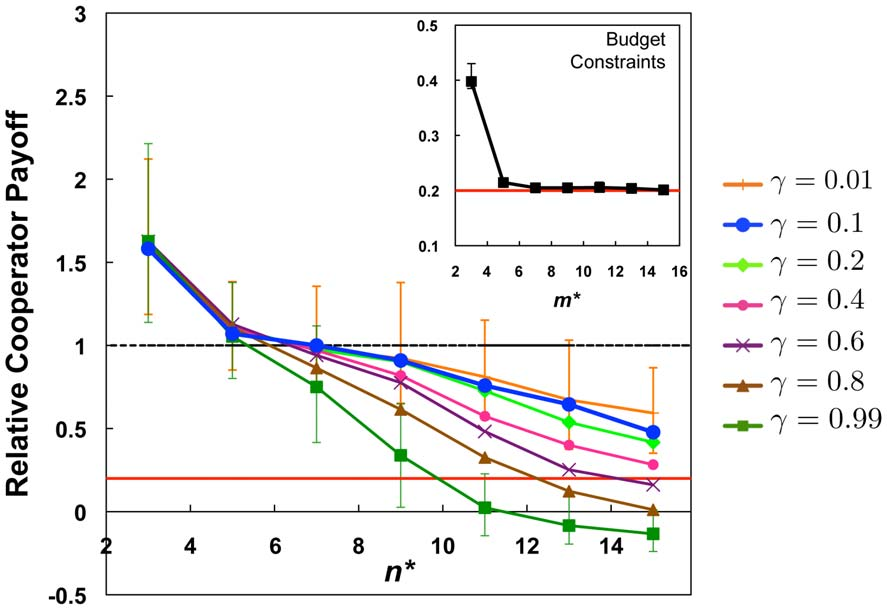
Relative cooperator payoff at equilibrium under capacity constraints for varying values of 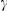, and budget constraints for 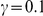 (inset). Only capacity constraints allow cooperators to outperform defectors, the threshold for which is indicated by the blue line at unity. The red line indicates the single game attractor for the unconstrained model. Here, 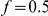, 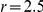, 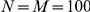. Values are averaged from 100 runs, error bars are 95% confidence intervals.

Our results focus on the *relative cooperator payoff*, defined as the average payoff to cooperators divided by the average payoff to defectors at equilibrium. Cooperators have an advantage when the relative cooperator payoff is greater than one. Without constraints (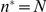, 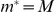) the model resolved to an equilibrium in which all agents were playing all remaining active games. The payoffs were equivalent to a single public goods game, in which cooperators are heavily exploited by defectors. Figure 2 shows the relative cooperator payoff at equilibrium under capacity constraints and budget constraints. Severe budget constraints allowed cooperators to do slightly better than in the unconstrained model due to defectors' inability to invade all games, but cooperators still dramatically underperformed relative to defectors. Capacity constraints, on the other hand, allowed cooperators to outperform defectors, but this advantage decreased as the maximum group size increases.

Figure 2 also shows that the relative cooperator payoff was largely unaffected by changes in the propensity to join or leave, 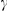, for low values of 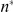, but as 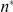 increased, cooperators did worse for larger values of 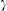. Larger 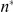 translated to more available games to join, and larger values of 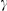 provided defectors with more opportunities to join available games with many cooperators before cooperators could leave the games that connected defectors to the larger world. When 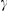 and 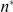 were large, the relative cooperator payoff could drop below the value for when all agents were in all games. This was due to negative assortment resulting from the high rate of leaving and joining combined with the constraint that individuals could only join games played by a current co-player. This is supported by the fact that more games remained active for large values of 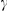 and 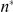, indicating that agents were more likely to be “stuck” in undesirable games. Our results were also robust for a wide range of system sizes. The relationship between 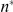 and relative cooperator payoff held for all the values we tested, between 50 and 500 agents (Figure S3A), though larger populations unsurprisingly took slightly longer to reach equilibrium (Figure S3B).

**Figure 3:**
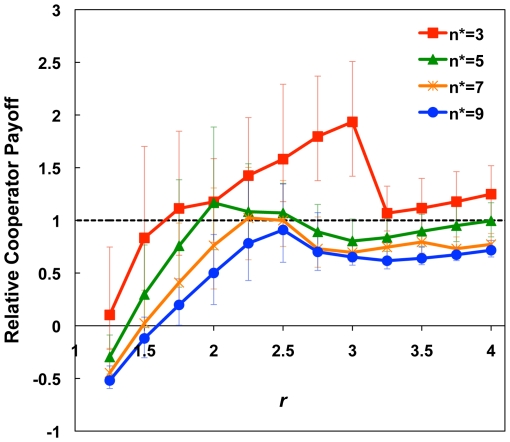
Relative cooperator payoff at equilibrium under capacity constraints for several values of the game production 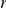. Here, 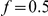, 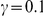, 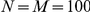. Values are averaged from 100 runs, error bars are 95% confidence intervals.

Figure 3 shows the relation between capacity constraints and game production, 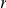, for 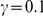. When 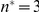, relative cooperator payoff increased with game production up to 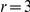. For 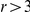, the game was no longer a social dilemma, since the best move was to cooperate even if the other two players defected. This led cooperators to stay in games in which all other players were defectors, and thus decreased their relative payoff. For 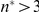, relative cooperator payoff did not increase monotonically with 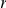, in contrast to many previous public goods models [8], [10], [11], [13], [16], [30]. This is because larger values of 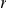 increased the overall agent payoff, leading to potential decreases in the minimum number of cooperators required for cooperators to stay in a game. Even though cooperators now did better in those games than with lower 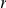, the fact that they stayed rather than left decreased their relative payoffs. In real systems, the equivalent of the game production will likely vary between institutions, further complicating analysis. Still, the take-home here is that larger returns on investments may also permit more freeloading [31].

**Figure 4:**
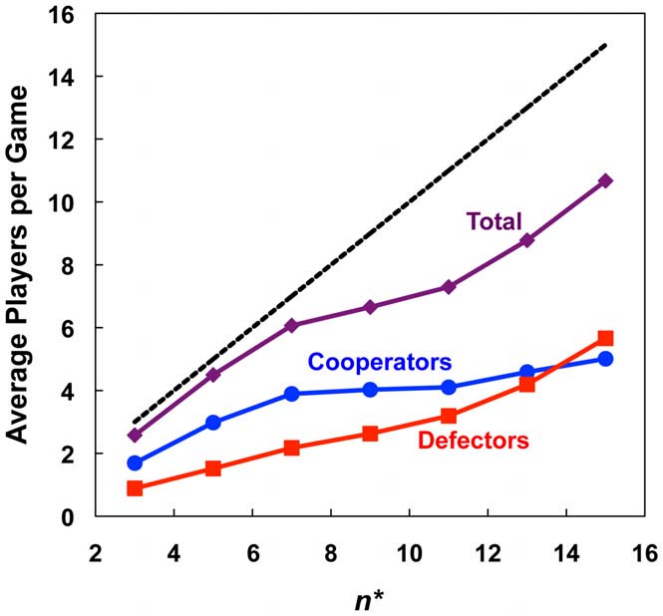
Average number of players per active game at equilibrium under capacity constraints. For 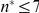, most games are full to capacity. For larger values of 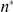, defectors are better able to infiltrate games, making fewer games desirable for cooperators.

The essential intuition of the capacity constraints result is that positive assortment is driven by the exiting process. Cooperators leave games with too many defectors, and stay in games with enough cooperators so that they get a positive return on their investment. Defectors, in turn, stay in games until all cooperators have left. With capacity constraints, defectors are unable to enter games with a cooperator majority, since no one will ever leave those games. Thus, at equilibrium, cooperators will mostly be found in games in which they constitute the majority. In games of this type that still contain defectors, however, defectors still outperform their co-players. This advantage to defectors, then, must be outweighed by the influence of cooperator-only games, in which cooperators receive their maximum payoff, and defector-only games, in which defectors receive zero payoff. When game sizes are limited, cooperators can cluster safely together, while defectors are occasionally stranded without any cooperators to exploit. This point is illustrated by Figure 4, which shows the average number of players per active game at equilibrium. For 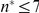, almost all games are at capacity. This corresponds to the maximum 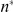 for which cooperators outperform defectors on average (Figure 2). For larger maximum game sizes, defectors more successfully occupy active games. As a result, fewer games are desirable for cooperators, which also leads to higher levels of cooperator flight and fewer active games at equilibrium (Figure S2).

### Cooperator Frequency and Excess Assortment

**Figure 5:**
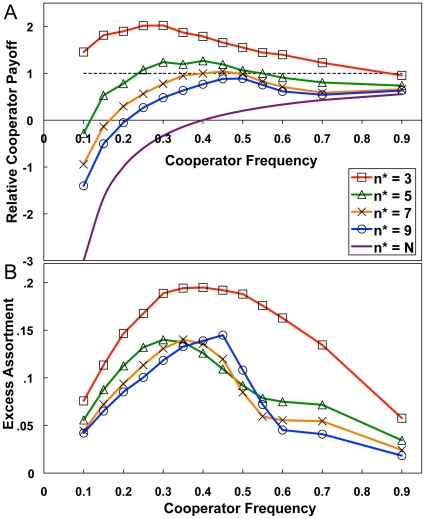
Average relative cooperator payoff (A) and excess assortment (B) at equilibrium as a function of the global cooperator frequency. Cooperator success is highly correlated with the ability of cooperators to assort at a level beyond that predicted by chance.

**Figure 6:**
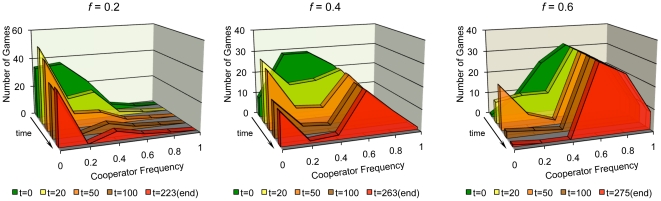
Example time courses under capacity constraints (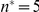) showing the frequency of within-game cooperator frequencies for several values of 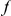. Cooperators tend to leave games where they are in the minority. Overall cooperator success, where the relative cooperator payoff is greater than one, is a balancing act in which the games in which they are exploited by defectors are countered by their success in cooperator-only games as well as the loss incurred by defectors in defector-only games.

In our model, the frequency of strategies does not change. Hence, the global frequency of cooperators, 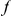, is a free parameter in the model. Figure 5A shows the relative cooperator payoff under capacity constraints for varying values of 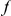. The payoff advantage to cooperation was not a monotonic function of global cooperator frequency: cooperators did best, relative to defectors, when in the minority, at frequencies close to 0.4 (Figure 5A). This can be explained by considering the model dynamics. Cooperators flee games in which defectors have too strong a presence. If there are enough games available, cooperators will only be found in games where they have a majority presence. In such games, any defectors will still be able to exploit the cooperators. Thus, cooperators only win out in the end if the games in which they are exploited are outweighed by the effects of the cooperator-only games and the defector-only games. Figure 6 illustrates this point by showing the number of active games with each possible within-game cooperator frequency for some example runs with 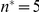.

Cooperators tended to do best when there were enough defectors in the population to force the defectors into many defector-only games, where defectors received zero payoff. Maximizing both cooperator-only and defector-only games is equivalent to maximizing the degree of positive assortment in the population, the extent to which like strategies tend to cluster together. A useful measure [32] for this is the *excess assortment*, 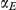, which is the average observed assortment,
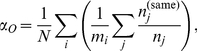
(2)minus the expected assortment,
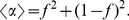
(3)Here, 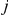 sums over all games played by individual 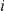, and 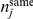 is the number of individuals in game 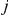 who play the same strategy as 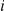.

**Figure 7:**
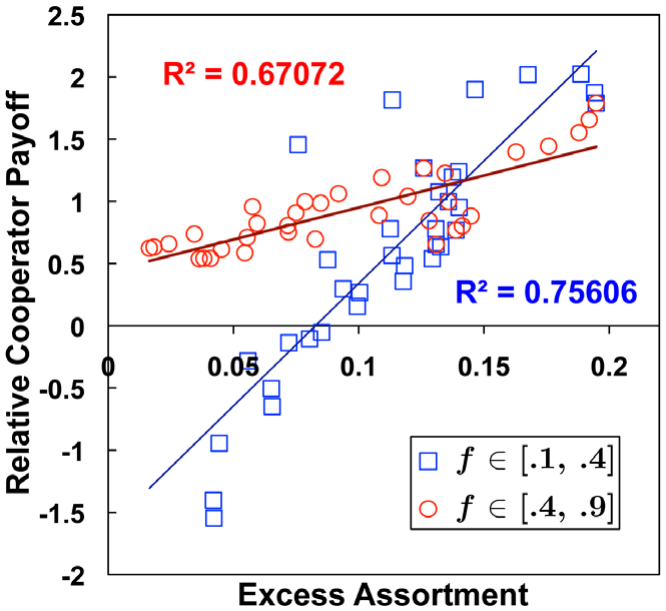
Relative cooperator payoff is highly correlated with the ability of agents to assort by type. This scatterplot relates the averages from across all tested cooperator frequencies, for a range of capacity constraint conditions (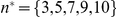). Both excess assortment and relative cooperator payoff increase with cooperator frequency up to about 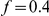 and then decrease. Coupling between excess assortment and relative cooperator payoff is basically linear both above and below this threshold frequency, but at different rates.

Figure 5B shows that the excess assortment was maximal when cooperators represented slightly less than half the population, and that excess assortment corresponded quite well to the relative cooperator payoff. Further analysis showed strong correlations between excess assortment and relative cooperator payoff on either side of the maximum assortment (Figure 7). We ran linear regressions on the aggregate results from 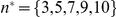, and found 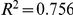 (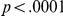) for 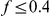 and 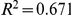 (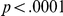) for 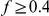. Some additional sensitivity analyses may be found in the Supporting Information (SI) Text S1, and in Supporting Figures S1, S2, S3, and S4.

## Discussion

As a broad generalization, network and institutional structures that endogenously produce positive assortment will promote cooperation [33]. As we consider more scenarios with multiple social games and endogenous and dynamic network structures, an important question can be phrased as: How can we get positive assortment in an ecology of games?

Our model suggests that capacity constraints are one effective mechanism for producing positive assortment and increasing cooperation in an ecology of games, while individual budget constraints are not sufficient to promote cooperation. Previous work [32] has shown that positive assortment can occur when both types of actors flee defector-heavy patches, and that limitations on patch capacity promote positive assortment. We show this to be the case in the context of an ecology of public goods games, and that these mechanisms may be used to promote cooperation.

Capacity constraints allow cooperators to assort and prosper in the absence of complex individual or institutional machinery. Such simple institutions may be preferred over more complex institutions with high transaction costs [34], [35], including sanctioning mechanisms[36]. However, incorporating more complex strategies, network processes, and institutions into the ecology of games framework will be an important area for future study [4], [9], [19], [21], [37], [38] (see Text S1). From a comparative institutional analysis perspective, it is important to analyze the effectiveness of different types of institutions for promoting cooperation, especially when transaction costs vary across institutional arrangements.

These findings have important implications for social and political organization in complex societies. Political theories of democracy promote inclusiveness as a normative goal. The norm of inclusiveness is also being promoted in many modern policy processes that rely on “collaborative” decision-making [2], [39], [40]. But the requirement of positive assortment creates a challenge for the promotion of cooperation, because it relies on the *exclusion* of defecting strategies and selective sorting, rather than extending invitations to everyone. Such empirical implications are, of course, recognized in social animals that use ostracism. Our findings suggest further empirical research on positive assortment mechanisms in real-life settings. Informal norms that promote positive assortment may conflict with formally promoted principles of inclusiveness in democratic societies.

In the course of a run of our model, games and individuals may become isolated. Over a longer time scale than that considered here, isolated groups of individuals would compete with one another [15]–[17]. The consideration of evolutionary dynamics, in which the relative fitness of isolated groups are compared, will be an important area for future research.

It crucial to recognize that political and social institutions rarely exist in isolation. Outcomes in nearly every social system are a product of individual decisions in multiple games, and analyzing one game at a time risks incorrect inferences. Our model of cooperation in an ecology of games pits the individual's interests in one game not only against those of the other players in that game, but also against those of the individuals in all his other games, *including himself*. In the model presented here, the agents are very simple, playing pure strategies without adaptation, memory, or other complex mechanisms. These features can clearly be elaborated upon. More importantly, the model provides a framework for the theoretical analysis of a variety of processes in an ecology of games beyond the public goods game. Models that consider the ecology of games are vital for advancing our understanding of complex political and social processes.

## Supporting Information

Text S1 Additional analyses.
(PDF)

Figure S1 Average number of games at equilibrium under capacity constraints for several values of γ. As defectors can more easily follow cooperators games and so by chase them out, the number of active games decreases. For these runs, *f* = 0∶5, *M* = *N* = 100, *r* = 2∶5.
(TIF)

Figure S2 The more games there were initially, the greater an opportunity agents had to drop them. This was particularly the case for low values of n*, where the ability to keep defectors out of cooperator-heavy games was most present.
(TIF)

Figure S3 Effects of system size on relative cooperator payoff (A) and average time to equilibrium (B). For these runs, *N = M*. Our results scaled very well, being little effected by a change in system size. For small systems, when *N = M* = 50, the system occasionally exhibited long transient cycles, with some cooperators continuously leaving and joining games in response to realized payoffs, and leading to an increase in time to equilibrium in the data (B).
(TIF)

Figure S4 Relative cooperator payoff was unaffected by the population size, as long as the number of available games changes correspondingly.
(TIF)
