# Supplementary material for: An Institutional Mechanism for Assortment in an Ecology of Games
Source: PLoS One. 2011 Aug 5;6(8):e23019. doi: 10.1371/journal.pone.0023019 (PMC3151282; doi:10.1371/journal.pone.0023019)
Supplement: Text S1 — Additional analyses. (PDF) [file pone.0023019.s001.pdf]

# Supporting Information (SI) for An Institutional Mechanism for Assortment in an Ecology of Games

Paul E. Smaldino[1], Mark Lubell[2,*]
**1 Department of Psychology, University of California-Davis, Davis, CA, USA**
**2 Department of Environmental Science and Policy, University of California-Davis, Davis, CA, USA**
**∗ E-mail: pesmaldino@ucdavis.edu**

## Relationships with Other Mechanisms that Promote Cooperation and/or Positive Assortment

Our model uses agents that are cognitively and behaviorally simple. Previous models have shown how implementations of certain cognitive and/or behavioral mechanisms can facilitate positive assortment by cooperators. These include:

- **Punishment**. If individuals can spend resources to punish noncooperators, cooperation can be evoked in dynamic individuals, or evolve more easily in populations [1–4].
- **Reputation**. The ability to record and remember who has cooperated and who has defected can promote cooperation [5–7], though this mechanism may break down if defectors can easily find new naive cooperators to exploit [8]. Reputation can also interact with punishment, as a reputation for nonpunishment can leave an individual vulnerable to exploitation [3]. Individuals may learn In considering an ecology of games, future models may include a mechanism for the admittance of new members based on reputation.
- **Defector aversion**. A tendency to leave patches heavy in defectors can leave to positive assortment [9]. In addition, in some cases, the tendency to simply 'walk away' from defecting opponents can promote the evolution of cooperative strategies [10,11], as can an attraction to neighborhoods more rich in cooperators [12].
- **Strategic reallocation**. In this model, resources are always divided evenly among all current games. A further refinement would be to allow agents to strategically allocate resources to games based on likelihood of future payoffs (i.e., local frequency of cooperators). This mechanism might either help or harm cooperators, depending on its implementation.

The structure of social networks can also influence the evolution of cooperation. For example, cooperation is enhanced on heterogenous (scale-free) networks, particularly when per-agent contributions are limited. [13]. Additionally, having fewer social ties (lower degree distributions) seems to enhance cooperation across network types [14].

While participation in multiple games is endogenous in our model, we do not explicitly provide a mechanism for the creation or destruction of games that is inherent in real-world ecologies of games. For example, political actors often create new policy venues and social actors create new groups. Our results suggest that any process of game creation that generates positive assortment will favor cooperation, while games that open participation to all strategies are vulnerable to predation by defectors.

## Active games and static equilibria

1. As $n^*$ increases, the number of active games decreases (Figure S1). When capacity constraints are relatively relaxed, defectors can easily join the most attractive games, i.e., those with relatively many

cooperators. This in turn makes those games less desirable for the cooperators, who flee. Consider the limiting case of the unconstrained model. Cooperators will continue to leave games where there are many defectors, but they will also join games experimentally. Defectors will join games and not leave unless all cooperators have fled. Eventually, all defectors will be in all active games. Cooperators will then re-join all remaining games, since their payoff increases if their contributions are divided among the most possible games. The unconstrained model seems to settle on a limit of six active games, which was the median and modal number of active games at equilibrium for $f = .5$, $r = 2.5$, $N = 100$. This result is insensitive to varying values of $M$.

2. The more games there are initially (i.e., for larger values of $M$), the lower the frequency of those games that will remain active at equilibrium. See Figure S2.

# Robustness with respect to population structure

We confirmed our results were robust over a wide range of system sizes, for $N = M$ between 50 and 500 (Figure S3). Larger population sizes tended to have a slightly higher relative cooperator payoff for large values of $M$, probably because of the increased number of initial games (Figure S4).

Having more initial games relative to population size also promoted cooperation, due to more chances for cooperator-heavy games. Likewise, too few initial games reduced the cooperator advantage (Figure S2). For example, with $f = 0.5$, the mean relative cooperator payoff for $n^* = 5$ dropped below unity around $M/N = 0.82$, though cooperators retained the advantage for all values of $M$ when $n^* = 3$. Thus, having enough options is vital for cooperator success. Our results also confirm findings that more available patches promoted positive assortment when individuals flee patches heavy with defectors [9].

# References

1. Boyd R, Richerson PJ (1992) Punishment allows the evolution of cooperation (or anything else) in sizeable groups. Ethol Sociobiol 13: 171–195.

2. Fehr E, Gächter S (2000) Cooperation and punishment in public goods experiments. Am Econ Rev 90: 980–994.

3. Brandt H, Hauert C, Sigmund K (2003) Punishment and reputation in spatial public goods games. Proc R Soc Lond B 270: 1099.

4. Hauert C, Traulsen A, Brandt H, Nowak MA, Sigmund K (2007) Via freedom to coercion: The emergence of costly punishment. Science 316: 1905–1907.

5. Milinski M, Semmann D, Krambeck HJ (2002) Reputation helps solve the 'tragedy of the commons'. Nature 415: 424–426.

6. Aktipis CA (2006) Recognition memory and the evolution of cooperation: How simple strategies succeed in an agent-based world. Adapt Behav 14: 239–247.

7. Skyrms B (2009) Groups and networks: Their role in the evolution of cooperation. In: Levin SA, editor, Games, groups, and the global good, Heidelberg: Springer. pp. 105–114.

8. Dugatkin LA, Wilson DS (1991) Rover: A strategy for exploiting cooperators in a patchy environment. Am Nat 138: 687–701.

9. Pepper JW (2007) Simple models of assortment through environmental feedback. Artif Life 13: 1–9.

10. Aktipis CA (2004) Know when to walk away: Contingent movement and the evolution of cooperation. J Theor Biol 231: 249–260.

11. Santos FC, Pacheco JM, Lenaerts T (2006) Cooperation prevails when individuals adjust their social ties. PLoS Comput Biol 2: 1284–1291.

12. Helbing D, Yu W (2009) The outbreak of cooperation among success-driven individuals under noisy conditions. Proc Natl Acad USA 106: 3680–3685.

13. Santos FC, Santos MD, Pacheco JM (2008) Social diversity promotes the emergence of cooperation in public goods games. Nature 454: 213–216.

14. Ohtsuki H, Hauert C, Lieberman E, Nowak MA (2006) A simple rule for the evolution of cooperation on graphs and social networks. Nature 441: 502–505.
