# Supplementary figures and images for: An Institutional Mechanism for Assortment in an Ecology of Games
Source: PLoS One. 2011 Aug 5;6(8):e23019. doi: 10.1371/journal.pone.0023019 (PMC3151282; doi:10.1371/journal.pone.0023019)

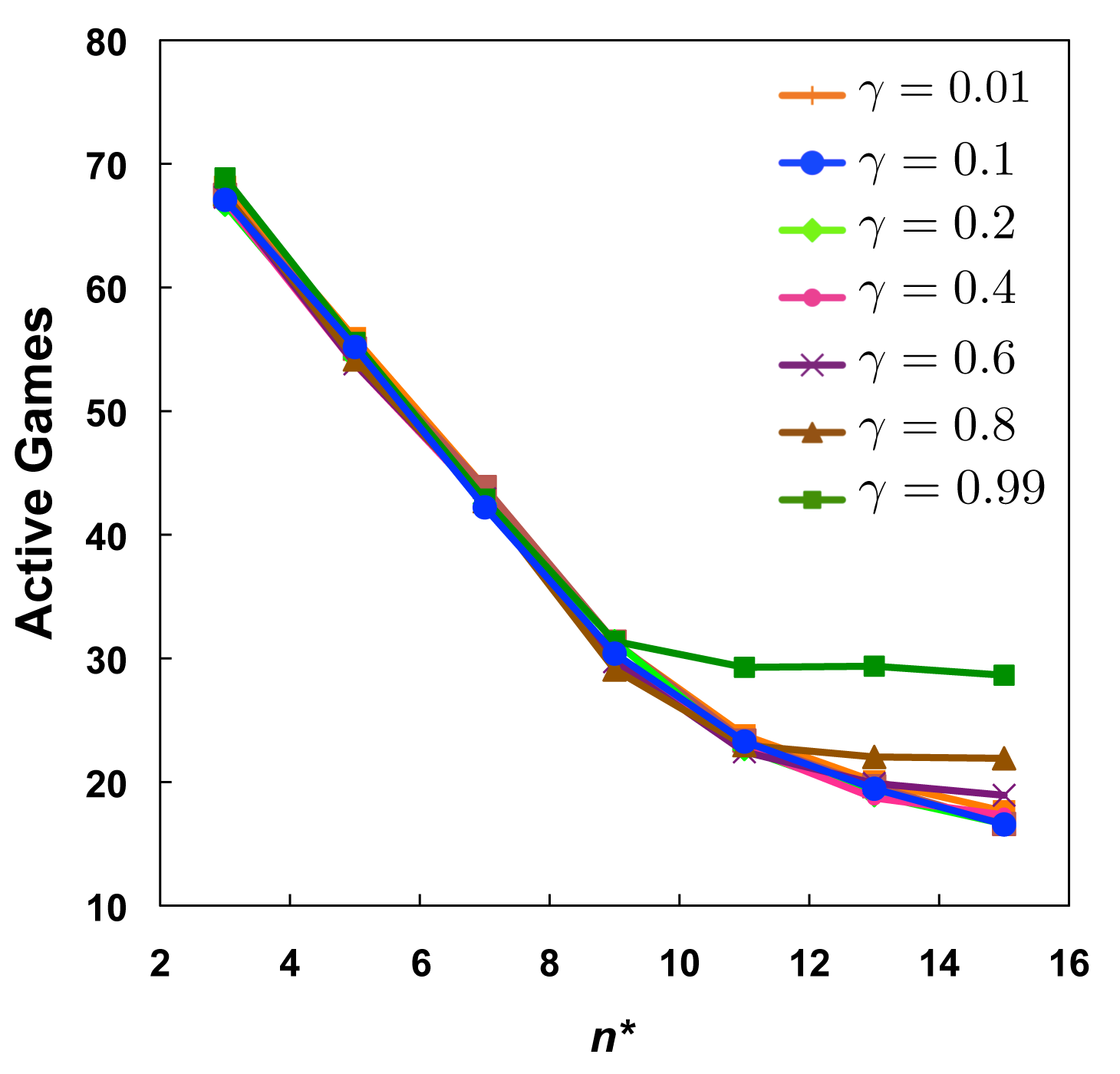

Supplement: Figure S1 — Average number of games at equilibrium under capacity constraints for several values of γ. As defectors can more easily follow cooperators games and so by chase them out, the number of active games decreases. For these runs, f = 0∶5, M = N = 100, r = 2∶5. (TIF) [file pone.0023019.s002.tif]

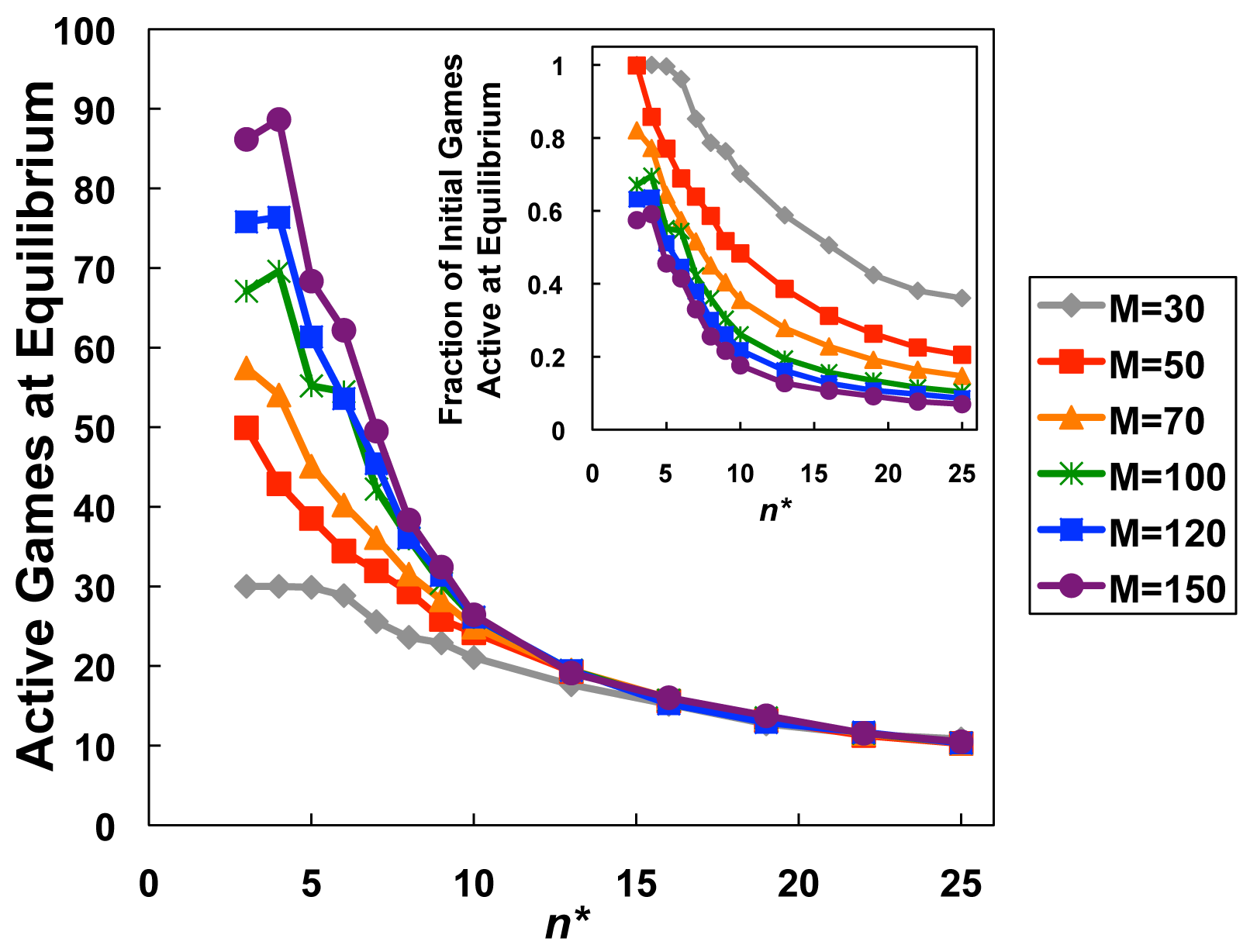

Supplement: Figure S2 — The more games there were initially, the greater an opportunity agents had to drop them. This was particularly the case for low values of n*, where the ability to keep defectors out of cooperator-heavy games was most present. (TIF) [file pone.0023019.s003.tif]

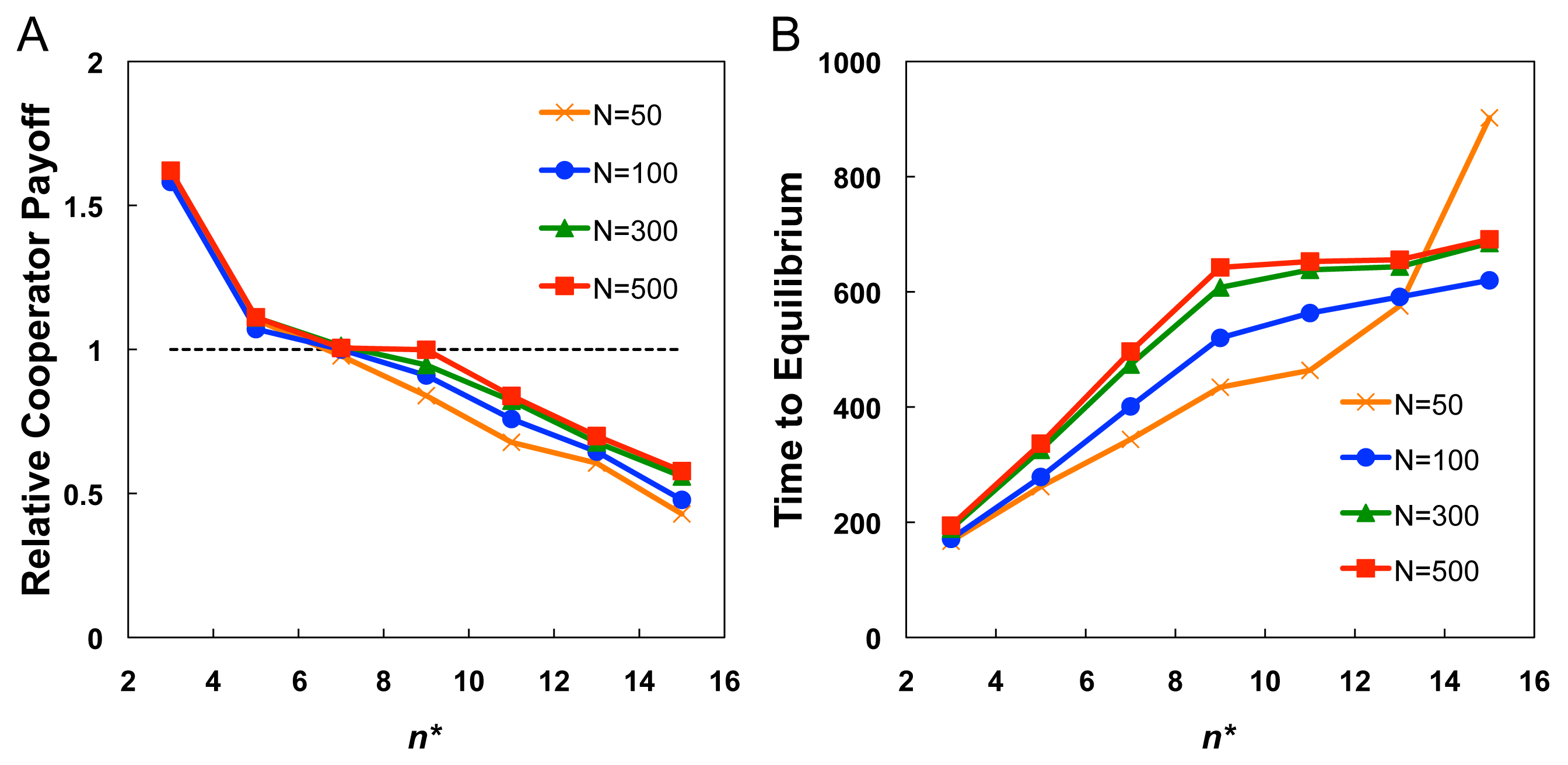

Supplement: Figure S3 — Effects of system size on relative cooperator payoff (A) and average time to equilibrium (B). For these runs, N = M. Our results scaled very well, being little effected by a change in system size. For small systems, when N = M = 50, the system occasionally exhibited long transient cycles, with some cooperators continuously leaving and joining games in response to realized payoffs, and leading to an increase in time to equilibrium in the data (B). (TIF) [file pone.0023019.s004.tif]

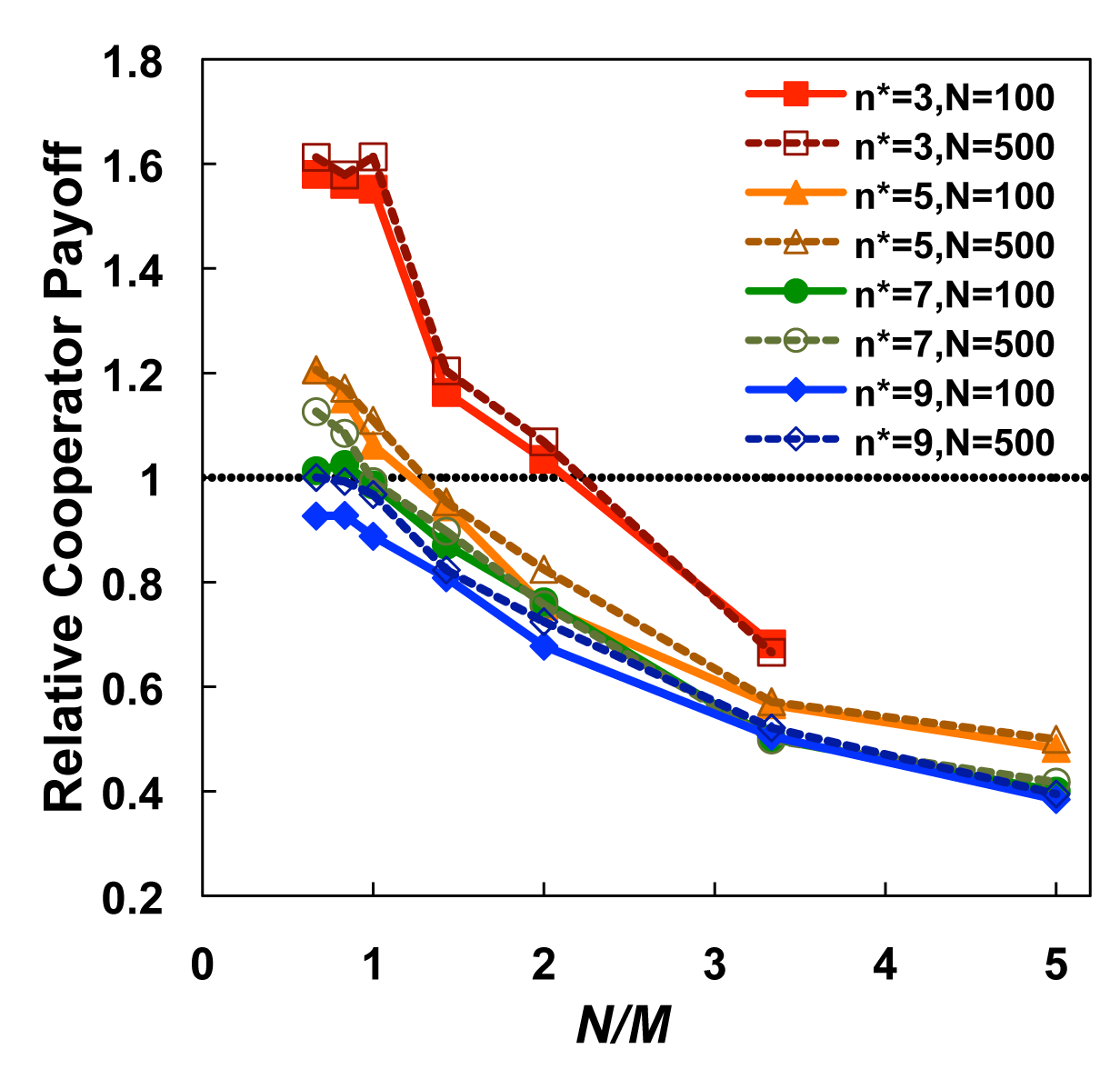

Supplement: Figure S4 — Relative cooperator payoff was unaffected by the population size, as long as the number of available games changes correspondingly. (TIF) [file pone.0023019.s005.tif]
